# Directly Acting Antiviral-Based Treatment for HCV-Infected Persons Who Inject Drugs: A Multicenter Real-Life Study

**DOI:** 10.3390/life11010017

**Published:** 2020-12-30

**Authors:** Vincenzo Messina, Lorenzo Onorato, Giovanni Di Caprio, Ernesto Claar, Vincenzo Iovinella, Antonio Russo, Valerio Rosato, Angela Salzillo, Riccardo Nevola, Filomena Simeone, Fabio Curcio, Mariantonietta Pisaturo, Nicola Coppola

**Affiliations:** 1 Infectious Diseases Unit, AORN Sant’Anna e San Sebastiano, 81100 Caserta, Italy; vincenzomessina.ce@libero.it (V.M.); lorenzoonorato@libero.it (L.O.); giov.dicaprio@gmail.com (G.D.C.); antoniorusso.ar.ar@gmail.com (A.R.); angelasalzillo.md@libero.it (A.S.); nucciasimeone@libero.it (F.S.); mariantonietta.pisaturo@unicampania.it (M.P.); 2 Hepatology Unit, Evangelical Hospital Betania, 80147 Naples, Italy; ernestoclaar@gmail.com (E.C.); valerio.rosato@libero.it (V.R.); riccardo.nevola@unicampania.it (R.N.); 3 Department of Internal Medicine, San Paolo Hospital, ASL Napoli 1 Centro, 80145 Naples, Italy; vincenzoiovinella56@gmail.com; 4 Department of Mental Health and Public Medicine, Section of Infectious Diseases, Second University of Naples, 80131 Naples, Italy; 5 UOC Dipendenze Ovest, ASL Napoli 1 Centro, 80145 Naples, Italy; curcio.fabione@gmail.com

**Keywords:** PWID, DAA, HCV infection, HCV chronic hepatitis, HCV treatment, Interferon-free, HCV failure, SVR, drug users, antiviral therapy

## Abstract

Background: We aimed to evaluate the factors associated with a virological response in a cohort of Hepatitis C virus (HCV)-infected people who inject drugs (PWID) treated with direct acting antivirals (DAAs). Methods: We conducted a multicenter retrospective cohort study enrolling HCV-infected PWID treated with DAAs. The primary outcome evaluated was the sustained virological response (SVR12) rate. Results: Five hundred and twenty HCV-infected PWID treated with all-oral DAA-based regimens were enrolled; a total of 168 (32.3%) patients presented genotype 1a, 109 (21.0%) genotype 1b, and 174 (33.5%) genotype 3; a total 152 of the 520 subjects (29.2%) were cirrhotics; a total 118 (22.7%) and 373 (71.7%) were treated with DAA regimens of second and third generation, respectively; a total 169 (33.6%) patients were receiving an opioid agonist at the start of antiviral therapy. Only 11 subjects (2.1%) did not show an SVR12. A significant correlation was found between treatment with opioid substitution therapy (*p* < 0.001), Human Immunodeficiency Virus (HIV) coinfection (*p* = 0.002), and treatment with first- or second-generation regimens (*p* = 0.0015) and HCV failure. Upon multivariate analysis, treatment with a first- or second-generation DAA was the only factor independently associated with failure (OR 10.4, 95% CI: 1.43 to 76.1, *p* = 0.02). Conclusions: Treatment with DAAs led to a high SVR12 rate (97.9%) in a large cohort of HCV-infected PWID. The only predictor of viral failure found in our analysis was treatment with first- and second-generation DAA.

## 1. Introduction

Hepatitis C virus (HCV) infection represents a global health problem, which affects 71 million people across the world and is responsible for about 400,000 deaths every year, according to World Health Organization (WHO) estimates [1]. Because of the high prevalence reported, people who inject drugs (PWID) have always been regarded as a key population to treat in order to achieve global elimination of HCV infection; indeed, it is estimated that about 52% of injection drug users are HCV-antibody positive [2]. 

HCV treatment of PWID has been demonstrated to be effective both at the individual level, preventing HCV-related morbidity and mortality [3], and at the population level, reducing the spread of infection and contributing to prevention of secondary cases [3,4,5]. However, in the interferon era, access to treatment for injection drug users has been limited by the high burden of adverse drug reactions and the insufficient compliance rates [6]. In recent years, the advent of oral directly acting antiviral (DAA) regimens has offered a highly effective and well tolerated therapy, leading to a substantial increase in treatment uptake in many countries [7]. However, only a few clinical trials have specifically addressed the efficacy and tolerability of DAAs in this special population [8,9,10], although subanalyses of experimental studies and data from real-world experience in subjects with recent injection drug use or taking opioid substitution therapy have reported compliance rates and treatment outcomes that were comparable to those of the general population [11,12]. Nevertheless, the uptake of HCV treatment is still suboptimal, because some clinicians may be reluctant to prescribe antivirals to patients with recent injection drug use or on opioid substitution therapy due to concerns about treatment adherence, drug–drug interaction and the risk of reinfection [13]. Thus, the few data on this topic come from real-world studies.

The aim of the present study was to evaluate the virological response to DAA-based regimens in a real-world cohort of HCV-infected PWID.

## 2. Methods

### 2.1. Patients

This was a multicenter retrospective cohort study conducted in three liver units, two in Naples and one in Caserta, in southern Italy; these centers have cooperated in previous investigations, sharing the same clinical approach [14,15,16]. We retrospectively evaluated all HCV-infected subjects who reported recent (during the last 12 months) or former injection drug use and were treated with an interferon-free regimen according to national and international guidelines in one of the participating centers from January 2017 to March 2019. Patients treated with sofosbuvir plus ribavirin and those who refused to provide informed consent to participate in the study were excluded from the analysis. At enrolment, epidemiological (age, gender), clinical (stage of liver disease, presence of comorbidities, opioid agonist treatment), biochemical (blood cell count, full liver and renal function tests), serological (anti-human immunodeficiency virus; HIV), and virological (HCV viral load and genotype) characteristics were collected for each patient.

Sustained virological response (SVR12) was defined as HCV-RNA below the limit of detection at week 12 after stopping antiviral therapy. Patients that showed a virological reactivation after discontinuation of treatment were defined as relapsers. The patients who failed to reach undetectable viral load during therapy were defined as nonresponders. The patients who showed detectable HCV-NA during treatment after a previous negative result were defined as having a viral breakthrough. 

The staging of liver disease was assessed on the basis of a liver biopsy, evaluated according to the METAVIR score [17], or, if not performed, through transient elastography, or in the presence of unequivocal clinical, biochemical, and ultrasonographic signs of liver cirrhosis.

Treatments with sofosbuvir plus simeprevir were defined as first-generation regimens; those with sofosbuvir plus daclatasvir, sofosbuvir-ledipasvir, or ombitasvir-paritaprevir-ritonavir and dasabuvir as second-generation regimens; and those with glecaprevir-pibrentasvir, sofosbuvir-/velpatasvir, or grazoprevir-elbasvir as third-generation regimens. 

### 2.2. Outcomes

The primary outcome of the present study was to evaluate the SVR12 rate of all-oral DAA-based regimens in our cohort of HCV-infected PWID. The secondary outcome was to investigate the correlation between the virological response and the epidemiological, clinical, and virological characteristics of the patients.

### 2.3. Laboratory Methods

HBV serum markers were analyzed using commercial immunoenzymatic assays (Abbott Laboratories, North Chicago, IL, USA, for HBsAg, anti-HBs, and anti-HBc). The anti-HCV antibody was assessed using a third-generation commercial immunoenzymatic test (Ortho Diagnostic Systems, Neckargemund, Germany). Antibodies to HIV 1 and 2 were sought using a commercial ELISA (Abbott Lab., North Chicago, IL, USA). Liver biochemistry and routine analyses were performed by routine methods.

For the assessment of viral load, viral RNA was extracted from 140 μL measures of plasma samples using a microspin column (QIAmp RNA viral kit, Qiagen GmbH, Hilde, Germany). HCV-RNA was quantified by performing a real-time polymerase chain reaction in a Light Cycler 1.5 (Roche Diagnostics, Branchburg, NJ, USA) with a detection limit in plasma samples of 40 UI/mL. HCV genotypes were determined by HCV genotype LIPA assay (Bayer, France) following the manufacturer’s instructions.

### 2.4. Statistical Analysis

Continuous variables were summarized as mean and standard deviation or median and interquartile range, and categorical variables as absolute and relative frequencies. For continuous variables, the differences were evaluated by Student’s t test or Wilcoxon rank-sum test; categorical variables were compared by chi-square test or Fisher’s exact test when appropriate. Age, gender, and all variables that were associated with SVR12 with a *p* value < 0.1 at univariate analysis were included in a logistic regression model to identify independent predictors of virological response; for the multivariable analysis we used the Firth method, based on penalized likelihood. A *p* value < 0.05 was considered statistically significant. The analysis was performed using IBM SPSS version 21.0 (Armonk, NY, USA).

### 2.5. Ethics Statement

All procedures applied in the study were in accordance with international guidelines, with the standards on human experimentation of the local ethics committees, and with the Helsinki Declaration of 1975 and its later amendments. This study was approved by the Ethics Committee of the Azienda Ospedaliera Universitaria of the University of Campania Luigi Vanvitelli (481/2018). All patients provided their informed consent to participate in the study.

## 3. Results

### 3.1. Baselines Characteristics of Patients

During the study period, 520 HCV-infected PWID treated with all-oral DAA-based regimens were enrolled. The main demographical, clinical, biochemical, and virological characteristics of subjects at enrolment are summarized in Table 1. Most of the patients (456, 87.5%) were male; the mean age was 47.7 ± 9.3 years. The median viral load was 1.2 × 10^6^ (IQR 3.64 × 10^5^–3.5 × 10^6^); as expected, the most common genotypes were 1a (168 patients, 32.3%) and 3 (174 patients, 33.5%), followed by genotypes 1b (109, 21%), 2 (29, 5.6%), and 4 (24, 4.6%); a total 152 of the 520 subjects (29.2%) were cirrhotics; a total 169 subjects (33.6%) were receiving an opioid agonist at the start of antiviral therapy.

Nine patients (1.7%) were treated with a first-generation DAA regimen, 128 (24.6%) with a second-generation and 383 (73.7%) with a third-generation regimen.

### 3.2. Virological Response and Associated Characteristics

Eleven of the 520 patients (2.1%) failed to achieve SVR12: 8 of them presented a relapse within the 12 weeks after the end of treatment and 3 interrupted treatment before the scheduled time. The characteristics of the patients stratified according to virological response are shown in Table 2. No significant difference in the demographic, clinical, or virological characteristics was found between patients with or without SVR12. Compared to those who achieved SVR12, a higher proportion of patients in the virological failure group were cirrhotic (54.5% vs. 28.7%), although this difference did not reach statistical significance (*p* = 0.09); moreover, a significant correlation was found between treatment with opioid substitution therapy (OST) and viral failure (*p* = 0.012). Similarly, HIV coinfection was more frequent in patients who failed to achieve SVR12 (18.2% vs. 2.6%, *p* = 0.002). Finally, subjects treated with first- or second-generation regimens showed a higher rate of virological failure than those treated with third-generation DAAs (5.8% vs. 0.8% respectively, *p* = 0.0015).

Upon multivariable logistic regression analysis, the treatment received was the only factor independently associated with the virological outcome (Table 3); in fact, subjects that were treated with a first or second-generation regimen had a higher likelihood of treatment failure than those treated with third-generation antivirals (OR 10.4, 95% CI: 1.43 to 76.1, *p* = 0.02; Table 3).

## 4. Discussion

In the present study, we evaluated the SVR12 rate to DAA-based treatment in a large cohort of HCV-infected PWID and analyzed the factors associated with virological failure.

It was estimated that in 2015, about 23% of all new cases of HCV infection occurred in people who inject drugs [1]. Both European [18] and US [19] guidelines recommend the implementation of screening and linkage to care programs, as well as harm-reduction interventions in order to limit the spread of infection among this population and to achieve the goals for HCV elimination proposed by the WHO [20]. A recently published study conducted by our group demonstrated that an innovative approach for screening and treatment based on close collaboration between infectious diseases physicians and facilities for substance-use disorders can significantly improve the uptake of antiviral treatments in these patients [21]. However, in many countries, access by PWID to effective therapy is limited by restrictions for reimbursement of DAA [22,23]. Moreover, even where treatment in this population is prioritized, prescribers may prefer not to offer the therapy to subjects reporting recent illicit drug consumption. In fact, one of the most important concerns of prescribers is the compliance of patients to treatment. However, a recent analysis [24] of the data reported for two multicenter phase 4 trials enrolling subjects with recent injection drug use or receiving opioid agonist therapy, who were treated with velpatasvir-sofosbuvir (SIMPLIFY-1) [10] or paritaprevir-ombitasvir-ritonavir plus dasabuvir (D3FEAT) [25], demonstrated that 184 of the 190 patients included (97%) completed the treatment, with a median adherence rate of 92%; recent stimulant injecting, unstable housing, and twice-daily regimens were associated with lower compliance. However, some clinicians may argue that it would be difficult to replicate the results derived from trials to clinical practice. In our cohort, 517 of the 520 patients (99.4%) completed the treatment, in line with the results observed in other real-life studies [26,27]. Because of the retrospective nature of the study, it was not possible to systematically assess the adherence of patients; however, the high SVR12 rate probably suggests acceptable compliance to treatment. Interestingly, no significant correlation between common predictors of failure reported in the literature and virological response was found in our study. In a large cohort study [27] of 934 Spanish PWID treated with DAAs, genotype 3, HIV coinfection, and liver cirrhosis were independently associated viral failure, as reported in the general HCV-infected population [28]. In our study, the SVR rates were similar between patients with genotype 3 and other genotypes (97.1% vs. 98.3%, *p* = 0.81); similarly, a nonsignificant difference in the response rate was found between cirrhotic and noncirrhotic patients (96.0% vs. 98.6%, *p* = 0.09). This observation could probably be explained by the fact that most of the patients were treated with highly effective, pangenotypic third-generation drugs. In fact, the only independent predictor of virological failure found in our study was the treatment received, most probably because of the higher antiviral potency and genetic barrier of the third-generation compared to first- and second-generation regimens, as well as their once daily dosing. A pooled analysis including the data of eight phase 2 or 3 trials showed that 151 of the 157 subjects receiving OST who were treated with glecaprevir-pibrentasvir obtained SVR12, and only one patient presented a viral failure [29]. Similarly, in the SIMPLIFY trial [10], among 103 subjects reporting recent injection drug use who were treated with sofosbuvir-velpatasvir, no virological failure was observed; the 6 patients who failed to achieve the SVR-12 were lost lo follow-up or presented a reinfection. Unfortunately, a long term follow-up was not available for most of the patients in our study, so we do not have data on reinfection rates.

Most importantly, the association between opioid substitution therapy and treatment failure found at univariate analysis was not confirmed by the multivariable logistic regression model. Several studies conducted on PWID have demonstrated a correlation between treatment with opioid agonists and the SVR rate to DAAs [27,30,31]. However, we should point out that the higher treatment failure in these studies was mainly due to a higher rate of losses to follow-up, and most of them included treatments that are now considered suboptimal. A recently published meta-analysis [12] including 38 observational or experimental studies for a total of 3634 patients demonstrated that people receiving OST and those reporting recent drug use achieved similar SVR12 rates compared to former drug users. 

## 5. Conclusions

Our study reports a high SVR12 rate in a cohort of HCV-infected injection drug users treated with interferon-free regimens. The only predictor of viral failure found in our analysis was the treatment received. However, further data from real-life studies, including recent drug users and subjects receiving OST treated with third-generation regimens, are needed to assess their efficacy and tolerability in this difficult-to-treat population.

## Figures and Tables

**Table 1:** Demographic, virological, and clinical data of the 520 patients enrolled.

No Patients	520
**Mean age (SD), years**	47.7 (9.3)
**Males,** ***n*** **(%)**	456 (87.5)
**HCV-RNA, median (IQR), UI/mL**	1.2 × 10^6^ (IQR 3.64 × 10^5^–3.5 × 10^6^)
**HCV genotype,** ***n*** **(%)**	
- **Genotype 1a**	168 (32.3)
- **Genotype 1b**	109 (21.0)
- **Genotype 2**	29 (5.6)
- **Genotype 3**	174 (33.5)
- **Genotype 4**	24 (4.6)
- **Mixed genotypes**	8 (1.5)
- **Unknown**	8 (1.5)
**HIV coinfection,** ***n*** **(%)**	16 (3.1)
**Staging of fibrosis (Metavir),** ***n*** **(%)**	
- **F0/F1**	130 (25.0)
- **F2**	88 (16.9)
- **F3**	105 (20.2)
- **F4**	152 (29.2)
- **Unknown**	45 (8.7)
**AST (xULN), mean (SD)**	1.48 (1.3)
**ALT (xULN), mean (SD)**	1.95 (1.8)
**Creatinine (mean, SD), mg/dl**	0.87 (0.96)
**Patients treated with OST,** ***n*** (%) *	169 (33.6)
***N*** **(%) pts treated with DAAs of**	
- **First generation**	9 (1.7)
- **Second generation**	128 (24.6)
- **Third generation**	383 (73.7)
**Length of treatment, (median, IQR), weeks**	12 (8–12)

Footnotes: ULN: Upper Limit of Normal; OST: Opioid substitution therapy. * Data available for 503 patients.

**Table 2:** Demographic, virological, and clinical data of the 520 patients enrolled, according to the response to DAA.

	SVR	no-SVR	*p* Value
**No patients**	509	11	
**Mean age (SD), years**	47.8 (9.3)	46.5 (8.4)	0.62
**Males,** ***n*** **(%)**	446 (87.6)	10 (90.9)	0.37 *
**HCV RNA, median (IQR), UI/mL**	1.2 × 10^6^ (3.68 × 10^5^–3.43 × 10^6^)	1.36 × 10^6^ (3.0 × 10^5^–3.45 × 10^6^)	0.41
**HCV genotype,** ***n*** **(%)**			
- **Genotype 1a**	166 (32.6)	2 (18.2)	0.81
- **Genotype 1b**	107 (21.0)	2 (18.2)	
- **Genotype 2**	28 (5.5)	1 (9.1)	
- **Genotype 3**	169 (33.2)	5 (45.4)	
- **Genotype 4**	24 (4.7)	0 (0.0)	
- **Mixed genotypes**	8 (1.6)	0 (0.0)	
- **Unknown**	7 (1.4)	1 (9.1)	
**Staging of fibrosis (Metavir),** ***n*** **(%)**			
- **F0/F1**	129 (25.3)	1 (9.1)	0.09
- **F2**	88 (17.3)	0 (0.0)	
- **F3**	101 (19.8)	4 (36.4)	
- **F4**	146 (28.7)	6 (54.5)	
- **Unknown**	45 (8.8)	0 (0.0)	
**HIV coinfection,** ***n*** **(%)**	13 (2.6)	2 (18.2)	0.03 *
**AST (xULN), mean (SD)**	1.48 (1.3)	1.47 (0.3)	0.95
**ALT (xULN), mean (SD)**	1.95 (1.8)	2 (0.5)	0.88
**Creatinine (mean, SD), mg/dL**	0.87 (0.97)	0.73 (0.2)	0.16
**Opioid substitution therapy,** ***n*** (%) **			
- **Patients receiving OST**	163 (31.3)	6 (54.5)	<0.001
- **Patients not receiving OST**	332 (63.8)	2 (18.2)	
- **Unknown**	14 (2.7)	3 (27.3)	
***N*** **(%) pts treated with DAA of**			
- **First generation**	9 (1.7)	0 (0.0)	0.0015
- **Second generation**	120 (23.6)	8 (72.7)	
- **Third generation**-	380 (74.7)	3 (27.3)	
**Length of treatment, (median, IQR), weeks**	12 (8–12)	12 (12–12)	0.48

Footnotes: OST: opioid substitution therapy, * *p* values calculated using Fisher’s exact test; ** Data available for 503 patients; *p* values < 0.05 are displayed in bold format.

**Table 3:** Variables independently associated with treatment failure upon logistic regression analysis.

Variables	OR	95% CI		*p* Value
		Lower	Upper	
**Gender (M vs. F)**	1.59	0.10	25.2	0.74
**Age**	1.12	0.98	1.27	0.09
**Liver fibrosis (F0–F3 vs. F4)**	0.28	0.03	2.24	0.23
**HIV serostatus (negative vs. positive)**	0.20	0.02	2.02	0.17
**Treatment received (1st/2nd vs. 3rd generation regimen)**	10.4	1.43	76.1	0.02
**Opioid substitution therapy (non-OST vs. OST)**	0.29	0.04	1.88	0.19

Footnotes: M: Male; F: Female; HIV: Human immunodeficiency virus; OST: Opioid substitution therapy. *p* values < 0.05 are displayed in bold format.

## Abbreviations

Hepatitis C virus	HCV
interferon alpha	IFN
ribavirin	RBV
sustained virological response	SVR
directly acting antiviral agents	DAAs
people who inject drugs	PWID
World Health Organization	WHO
Service for Dependence	SerD
